# The assessment value of pathological condition of serum adiponectin and amylin in primary osteoporosis and its correlation analysis with bone metabolism indexes

**DOI:** 10.5937/jomb0-35877

**Published:** 2023-01-20

**Authors:** Xiao Jie Wang, Xue Bai, Ying Miu, Pan Chen, Pi Jun Yan, Chun Xia Jiang

**Affiliations:** 1 Southwest Medical University, Affiliated Hospital, Department of Endocrinology and Metabolism, Luzhou City, Sichuan Province, China

**Keywords:** primary osteoporosis, adiponectin, amylin, serum, bone metabolism index, the evaluation value of the pathological condition, correlation, primarna osteoporoza, adiponektin, amilin, serum, indeks metabolizma kostiju, vrednost procene patološkog stanja, korelacija

## Abstract

**Background:**

This paper explores the assessment value of pathological condition of serum adiponectin (APN) and amylin in primary osteoporosis (POP) and their correlation with bone metabolism indexes.

**Methods:**

From January 2019 to June 2021, 79 cases of POP patients were selected as the research objects. A test of the patients' bone density was conducted, and clinical grading of POP was via T value (normal, mild, moderate, severe). The analysis of the assessment value of pathological condition of serum APN and amylin for POP and their association with bone metabolism indexes in patients was performed.

**Results:**

APN and amylin in patients were declined with POP's aggravation. APN of 5.15 μg/mL or less and amylin of 15.38 pmol/L or less were risk factors influencing the aggravation of pathological condition of POP (*P*< 0 .0 5). The area under the curve (AUC) of combined detection of APN and amylin to assess the severity of POP was elevated vs. alone test of amylin (*P*< 0.05). 25-hydroxyvitamin D (25-(OH) D) and total type 1 procollagen amino-terminal propeptide (t-PINP) in patients were descended with the aggravation of pathological condition of osteoporosis (*P* < 0.05). At the same time, no distinct differences were presented in the three groups of type I collagen hydroxyl terminal peptide b degradation product (β-CTX) and N-terminal osteocalcin (N-MID) (P> 0.05). APN, amylin, 25-(OH)D, β-CTX, and t-PINP were negatively linked with POP clinical grade (*P*< 0.05). APN and amylin were associated with 25-(OH) D, β-CTX, t-PINP (*P*< 0.05), and APN and amylin were not linked with N-MID (*P*> 0.05).

**Conclusions:**

Serum APN and amylin are provided with evaluation values for the severity of POP and are associated with bone metabolism in patients.

## Abstract (sr)

**Uvod:**

Ovaj rad ispituje značaj serumskog adiponektina i amilina kod procene patološkog stanja u primarne osteoporoze i analizu korelacije sa indeksima koštanog metabolizma.

**Metode:**

Kao predmet istraživanja, od januara 2019. do juna 2021. godine je odabrano 79 slučajeva pacijenata sa POP-om. Sproveden je test gustine kostiju pacijenata a klinička procena POP je urađena preko T vrednosti (normalna, blaga, umerena, teška). Urađena je analiza značaja procene patološkog stanja putem serumskog APN i amilina na POP i njihove povezanosti sa indeksima koštanog metabolizma kod pacijenata.

**Rezultati:**

APN i amilin kod pacijenata su smanjeni sa pogoršanjem POP-a. APN od 5,15 mg/mL ili manje i amilin od 15,38 pmol/L ili manje su bili faktori rizika koji utiču na pogoršanje patološkog stanja POP (P< 0,05). Površina ispod krive (AUC) kombinovane detekcije APN-a i amilina za procenu ozbiljnosti POP-a je bila povišena u odnosu na sam test amilina (P< 0,05). 25-hidroksivitamin D (25-(OH) D) i ukupni amino-terminalni propeptid prokolagena tipa 1 (t-PINP) kod pacijenata su sniženi sa pogoršanjem patološkog stanja osteoporoze (P< 0,05). U isto vreme, nisu predstavljene jasne razlike u tri grupe proizvoda b degradacije hidroksil terminalnog peptida kolagena tipa I (b-CTKS) i N-terminalnog osteokalcina (N-MID) (P> 0,05). APN, amilin, 25-(OH)D, b-CTKS i t-PINP su bili negativno povezani sa kliničkom ocenom POP (P< 0,05). APN i amilin su bili povezani sa 25-(OH) D, b-CTKS, t-PINP (P< 0,05), dok APN i amilin nisu bili povezani sa NMID (P> 0,05).

**Zaključak:**

APN i amilin u serumu su od značaja za procenu težine POP i povezani su sa metabolizmom kostiju kod pacijenata.

## Introduction

Primary osteoporosis (POP) is a systemic metabolic disease characterized by declined bone mass, bone fragility, susceptibility to fractures and destruction of bone tissue microstructure. Clinical data clarify patients shown bone metabolism disorders [1]. Relevant reports have elaborated that glucose and lipid metabolism disorder is also the crucial reason for POP except for age-triggered bone mineral density (BMD) loss [2], and research has manifested the BMD of lumbar vertebra and femur with aberrant lipid metabolism in patients is declined vs. the normal one, and a specific association of aberrant lipid metabolism with POP’s occurrence is further testified [3]. Adiponectin (APN), a specific protein secreted via fat cells, is available to augment insulin sensitivity and accelerate blood lipids. Studies have illuminated that APN can immediately work on bone, stimulate the proliferation and differentiation of human osteoblasts, and directly spur the nuclear factor-kB receptor activating factor ligand [4], elaborating that it might be associated with POP caused via aberrant bone metabolism. Amylin, constitutive of 37 amino acid residues, bears a resemblance to the physiological activity of calcitonin gene-related peptide, which is available to accelerate bone cell proliferation and restrain bone resorption. Nevertheless, the association of the two with POP is still in exploration. Consequently, the research is to determine the assessment value of pathological condition of serum APN and amylin for POP and their relevance with bone metabolism indexes, offering references for clinical evaluation of disease.

## Materials and methods

### Clinical data

From January 2019 to June 2021, 79 cases of POP patients were selected as the research objects (Table 1). Examination of BMD in patients was via the ultrasonic BMD meter, and clinical grading of POP was via adopting T [5] [6], T-1-1 was normal, T-1-2 was mild, T-2-3 was moderate, and T-3-4 was severe. The formula to calculate T-score was: T-score = (bone mineral density-reference bone mineral density)/reference standard deviation. The diagnosis of osteoporosis was performed according to the following criteria: more than 2.5 SD decrease in BMD compared with the healthy population. Based on the lumbar spine T-score, it is divided into mild, moderate, and severe. The specific grading standards are as follows: (1) T-1-1: Normal: bone mass reduction is between 1SD and 2.5SD; (2) T-1-2: Mild osteoporosis: bone mass reduction between 2.5SD and 3SD; (3) T-2-3 Moderate osteoporosis: bone mass reduction between 3SD and 4SD; (4) T-3-4: Severe osteoporosis: bone mass loss is at least 4SD, or -2.5SD and one or more fractures occur.

**Table 1:** Comparison of general data among the mild, the moderate and the severe.

Classification	The mild<br>(n=21)	The moderate<br>(n=40)	The severe<br>(n=18)	F/_X^2^_	P
Gender (Male)	13	25	11	0.010	>0.05
Age (years)	51.39±5.72	52.08±6.14	52.97±6.02	0.349	>0.05
BMI (kg/m^2^)	22.14±2.13	23.31±2.10	22.65±2.39	2.170	>0.05
Smoking history	7	12	5	0.147	>0.05
Drinking history	6	10	2	1.906	>0.05
Combination hypertension	3	6	2	0.160	>0.05
Combination diabetes mellitus	5	4	1	3.439	>0.05
Combination heart disease	3	5	1	0.830	>0.05
Maternal family history	5	12	3	1.202	>0.05
Fracture history	3	6	5	1.622	>0.05
Anemia	3	2	2	1.616	>0.05
Calcium (mmol/L)	2.31±0.12	2.26±0.13	2.09±0.11	17.871	<0.05
Phosphorus (mmol/L)	1.11±0.09	1.05±0.13	0.96±0.17	6.614	<0.05
PTH (pg/mL)	5.97±0.37	6.12±0.36	6.47±0.52	8.136	<0.05

There were no distinctive differences in general data among the four groups of patients (*P*>0.05). Written informed consent was obtained from all participants. The present study was approved by the Institutional Review Board of The Affiliate Hospital, Southwest Medical University.

### Inclusion criteria

(1) Meeting the diagnostic criteria of POP in Guidelines for the Diagnosis and Treatment of Primary Osteoporosis (2017) [7]; (2) Over 18 years old; (3) Complete clinical data.

### Exclusion criteria

(1) Patients with secondary osteoporosis; (2) Patients with aberrant organ function like combining heart and brain; (3) Patients with combining malignant tumors; (4) Patients with combining aberrant hematopoietic system; (5) Patients with combining other types of bone diseases; (6) Patients who had taken active vitamin D and calcium therapy within 1 month before enrollment.

### Methods

Serum APN and amylin detection: Collection of 3 mL fasting venous blood was conducted after diagnosis of patients, and separation of the serum was performed after centrifugation. The serum APN and amylin examination in patients were conducted adopting an RT-6000 automatic microplate reader (American Redu corporation), and the kit was applied (American millipore).

Bone metabolism index examination: Test of 25-(OH) D, t-PINP, N-MID, and β-CTX in patients wasperformed adopting Cobas e601 automatic electro-chemiluminescence immunoassay analyzer (German Roche).

### Observation indexes

(1) Comparison of serum APN and amylin in the mild group, the moderate and the severe to analyze the assessment value of pathological condition of patients’ serum APN and amylin for POP and their association with the progression of pathological condition. (2) Comparison of the bone metabolism indexes in the mild, the moderate, and the severe to analyze the correlation of bone metabolism index with serum APN and amylin in patients and the two with the clinical grade.

### Statistical analysis

Data processing was carried out with SPSS22.0 software. The manifestation of enumeration data was given as a percentage. The comparison of the differences between groups was via the exerting x^2^ test. The representation of measurement data was in (Σx ± S) after a standard test. The two-group comparison of differences was via adopting a t-test, and the comparison of the differences among the multiple groups was via exerting one-way ANOVA. The analysis of the assessment value of pathological condition of APN and amylin for POP was via adopting the ROC curve; Analysis of the association of APN, amylin, and the progression of a pathological condition of POP was via exerting logistic regression. Differences were considered to be significant at *P*<0.05.

## Results

### Comparison of APN and amylin in patients among the mild, moderate, and severe

**Figure 1:**
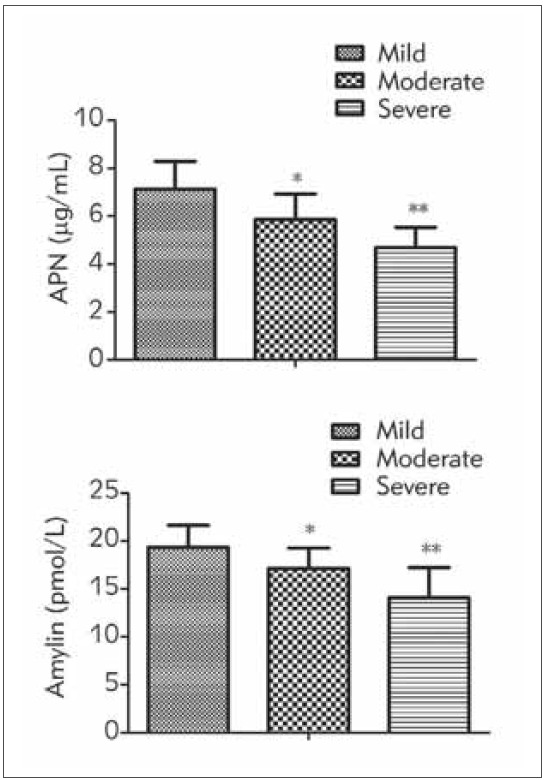
Comparison of APN and amylin in patients among the mild, the moderate, and the severe A comparison of APN and amylin in the three groups was conducted, and F was 27.377 and 24.190, separately, P<0.05.

RT-qPCR was performed to investigate the differential expression of APN and amylin in the mild, moderate, and severe POP. Compared with patients in the mild group, APN and amylin plasma levels were significantly lower in the moderate and severe groups. In addition, compared with the moderate group, plasma levels of APN and amylin were further reduced in the severe group. APN and amylin in patients were declined with POP’s aggravation (*P*<0.05), as manifested in Figure 1.

### Analysis of the evaluation value of pathological condition of APN and amylin for POP

**Figure 2:**
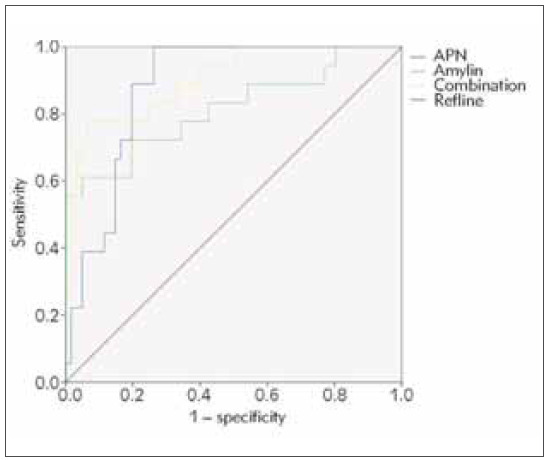
ROC curve analysis of APN and amylin to assess the severity of the pathological condition of POP.

ROC curves show that the AUC of APN and amylin in clinical samples for POP, when APN and amylin were combined, there was a much stronger evaluation value for POP (AUC = Area under the curve. AUC=0.903) than amylin (P<0.05) (Figure 2 and Table 2). The results suggest that APN and amylin could be used together to increase the evaluation value for POP.

**Table 2:** Analysis of the assessment value of pathological condition of APN and amylin for POP. Amylin vs. the combination, * *P*<0.05.

Indexes	Cut-off value	AUC	SE	95% CI
APN	5.15 μg/mL	0.879	0.037	0.806~0.952
Amylin	15.38 pmol/L	0.815^*^	0.066	0.686~0.945
Combination		0.903	0.041	0.824~0.983

The AUC of the joint test of APN and amylin to assess the severity of POP was elevated vs. alone detection of amylin (*P*<0.05), as presented in Table 1 and Figure 2.

### Logistic regression analysis of APN, amylin, and progression of a pathological condition of POP

Patients with APN of 5.15 μg/mL or less and amylin of 15.38 pmol/L or less were risk factors impacting the aggravation of pathological condition of POP (*P*<0.05), as manifested in Table 2.

### Comparison of bone metabolism indexes in patients among the mild, moderate, and severe

**Figure 3:**
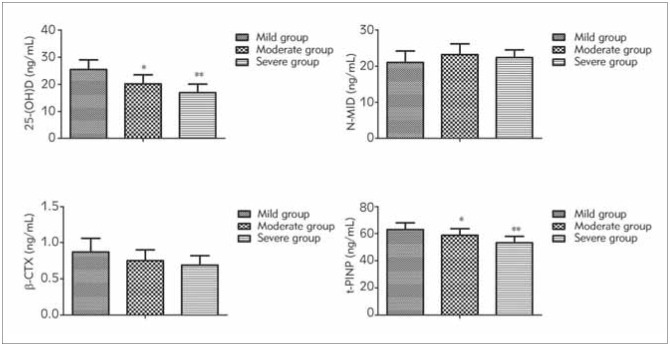
Comparison of bone metabolism indexes in patients among the mild, the moderate, and the severe comparison of 25-(OH) D, N-MID, β-CTX, and t-PINP in the three groups was performed, and F was 34.433, 4.068, 4.309, 20.059, PN-MID, β-CTX of more than 0.05, P_25-(OH) D_ and _t-PINP_ of less than 0.05.

25-(OH) D and t-PINP in patients were descended with the aggravation of pathological condition of POP (*P*<0.05); No distinct differences were presented in N-MID and β-CTX among the three groups (*P*>0.05), as manifested in Figure 3. Table 3

**Table 3:** Logistic regression analysis of APN, amylin and the progression of pathological condition of POP. Assignment: Severity of pathological condition (severe was 1, and mild and moderate were 0); APN (more than 5.15 μg/mL was 1, and 5.15 μg/mL or less was 0); amylin (more than 15.38 pmol/L was 1, and 15.38 pmol/L or less was 0).

Indexes	b	SE	wald X^2^	OR	95% CI	P
APN	-0.324	0.117	7.669	0.723	0.575~0.910	0.006
Amylin	-1.117	0.400	7.798	0.327	0.149~0.717	0.005
Constant term	1.402	0.332	17.833	4.063	2.120~7.789	<0.001

### Correlation analysis of APN, amylin, and bone metabolism indexes with POP clinical grades

**Figure 4:**
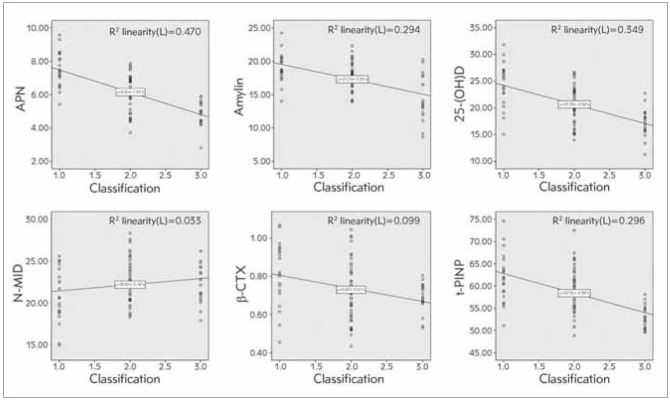
Association analysis of APN, amylin, bone metabolism indexes and clinical grades of POP.

APN, amylin, and 25-(OH) D, β-CTX, and t-PINP were negatively linked with POP clinical grades (*P*<0.05), as presented in Figure 4.

### Relevance analysis of APN, amylin with bone metabolism indexes

**Figure 5:**
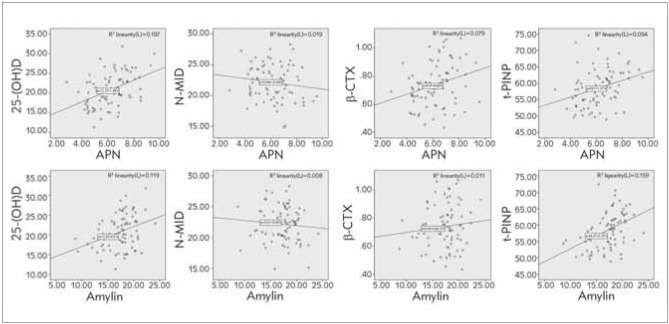
Correlation analysis of APN, amylin with bone metabolism indexes.

APN and amylin were associated with 25-(OH) D, β-CTX, and t-PINP (*P*<0.05), while there was no association between APN, amylin with N-MID (*P*>0.05), as manifested in Figure 5.

## Discussion

In the normal bone metabolism cycle, there is a dynamic balance between osteoblasts forming new bone and osteoclasts absorbing the old bone. Then bone reconstruction was carried out continuously. Bone resorption exceeds bone formation when this balance breaks, leading to POP [8] [9]. Relevant reports elucidate that the blood lipid metabolism disorder impacts the occurrence of POP [10]. Hyperlipidemia is available to lead to the elevation in the number of adipocytes in the bone marrow cavity and bone marrow microcirculation disorders, resulting in declined bone metabolism and the occurrence of POP [11] [12]. A novel cytokine pattern, ADP is an adipokine explicitly secreted via adipocytes. Its receptors exist in diversified tissues throughout the body. They are available to augment fatty acid oxidation and glucose uptake via impacting the insulin signaling pathway, thereby ameliorating the energy metabolism of bone cells. Amylin, a polypeptide composed of 37 amino acids secreted via pancreatic β-cells, is available to accelerate osteoblast proliferation and inhibit calcitonin-like osteoblast, thus influencing bone metabolism[13] [14]. Relevant studies have also elaborated that aberrant amylin can impact bone cell proliferation and metabolism [15]. The results of our research have shown that APN and amylin in patients decreased with POP aggravation, suggesting that APN and amylin in patients were linked with pathological condition of POP. Amylin can boost bone cell proliferation and its hyperplasia of osteoblasts in the active growth stage and delay bone absorption, thereby aggravating the severity of POP [16]. Additionally, we showed that the AUC of the combined detection of APN and amylin to assess the severity of POP was augmented vs. alone test of amylin, indicating that the combined detection has a higher evaluation value of POP severity. Therefore, it might be applied in the auxiliary assessment of the pathological condition of POP.

APN is available to promote hematopoietic stem cell proliferation via the MAPK signaling pathway and accelerate the differentiation and maturation of mesenchymal cells, inhibiting the differentiation of bone marrow preadipocytes into adipocytes, thereby modulating the balance of osteoblasts with adipocytes [17] [18]. Amylin can repress the activity of osteoclasts, decline the bone absorption of basal and parathyroid hormone, stimulate the generation of cAMP via combining with the calcitonin receptor on osteoclasts, and constrain the mutual fusion of osteoclast precursor cells for the formation of mature multinuclear giant cells. Therefore, osteocyte is suppressed to influence bone metabolism [19]. Our study showed APN of 5.15 μg/mL or less and amylin of 15.38 pmol/L or less in patients were risk factors impacting the aggravation of pathological condition of POP, elaborating that the declined serum APN and amylin in patients might aggravate the severity of the pathological condition of POP. The primary reason was that amylin was available to influence bone formation and activate protein kinase C to boost the proliferation of osteoblasts and chondrocytes. Aberrant amylin was able to result in bone metabolism disorders.

Bone metabolism indexes are available to reflect the change rate of bone metabolism, the function of osteoclasts and osteoblasts, and the frequency and rate of bone turnover [20]. Relevant reports have elaborated that bone metabolism in patients with osteoporosis is nearly linked to pathological condition severity [21] [22]. This research manifested 25-(OH) D, and t-PINP in patients descended from the aggravation of the pathological condition of osteoporosis, clarifying that bone metabolism disorders influenced POP. Bone marrow stromal cells are provided with bidirectional differentiation ability into osteoblasts and adipocytes. Osteoblasts can differentiate into adipocell-like cells via adding fatty acids to the culture system of osteoblasts. In contrast, the differentiation of bone marrow cells into osteoblasts is declined [23], which further illuminates the certain inevitable association of lipid metabolism with bone metabolism. This research elaborated that APN and amylin were linked with 25-(OH) D, β-CTX, and t-PINP, illuminating that aberrant serum APN and amylin in POP patients were associated with bone metabolism disorders and furthertestifying that blood lipid metabolism disorders and abnormal insulin secretion were linked with the occurrence of POP. The crucial reason was that declined amylin was available to weaken the activity of osteoblasts, strengthen the activity of osteoclasts, decline bone formation, aggrandize bone resorption, and then impact the aberrant bone metabolism.

In conclusion, the serum level of APN and amylin were provided with evaluation values for the severity of POP and were linked with bone metabolism in patients. Nevertheless, shortcomings were still in this research. The involvement of the small sample size might lead to biases in the study results. In the later stage of bone, the sample size should be elevated to analyze the association of serum APN, amylin, with POP.

## Dodatak

### Acknowledgments

Not applicable.

### Conflict of interest statement

All the authors declare that they have no conflict of interest in this work.
